# A Mentee's perspective on Dr. Anthony Monaco: the quiet giant of transplantation

**DOI:** 10.3389/frtra.2024.1375316

**Published:** 2024-02-27

**Authors:** Satish N. Nadig

**Affiliations:** ^1^ Feinberg School of Medicine, Northwestern University, Chicago, IL, United States; ^2^ Department of Surgery, Northwestern Medicine, Chicago, IL, United States; ^3^ Comprehensive Transplant Center, Northwestern Memorial Hospital, Chicago, IL, United States

**Keywords:** anti-thymocyte globulin, mentorship, leadership, transplant, immunology

## Abstract

A mentee's perspective of an academic journey on a path paved by a pioneering transplant surgeon-scientist.

On July 22, 2021, at 4:40 pm Eastern Standard Time, I received a touching and heartfelt email from Dr. Anthony Monaco. In this email, there was one line that epitomized his entire being.

“I am sure that you know that the best gift and reward a teacher and mentor can receive is the distinction achieved by their students”.

Anthony Monaco MD, FACS, was the founding director of the Harvard transplant service at Boston City Hospital that evolved into the Division of Transplant Surgery at Beth Israel Deaconess Medical Center, which bears his name today. Before this major feat, saw a man fiercely dedicated to his craft and the generally unaccepted idea of transferring tissues from one person to another. He and his contemporaries spent the greater part of the 1960s–2000s racing to find ways to evade the human immune system. Radical ideas of taking animal serum inoculated with that of humans and purifying the antibodies from this reaction to deplete effector T cells, set the foundation for the very immunosuppressant medications that we consider the “gold standard” today (1). His groundbreaking work started an entire field of medicine that, one could argue, is one of the most miraculous advancements in medicine of the last century. He rose the ranks to Division Chief and President of every important transplant society, including the American Society of Transplant Surgeons and The Transplantation Society. Yet, to me (and a score of others), these accomplishments are not what actually defines Tony. It is, in fact, the legions of mentees that he fostered along the way to make sure the field of Transplantation … his field, was kept in good stead and would progress beyond even his imagination. Although this tribute could delineate all of the tangible professional accomplishments that Tony achieved in his lifetime, of which there are many, I would rather take a moment and underscore the man and the mentor that Tony was.

It was the summer of 2003 and I had just matched to the Beth Israel Deaconess Medical Center as the first student from the Medical University of South Carolina to match in a surgical residency at a Harvard hospital. Insecurities abound. One of my very first rotations was on the very busy and demanding transplant service (a field I already knew I had an interest in.) It was during this first week that I met Dr. Monaco. With an intimate esophageal phonated “hello”, I realized that I was in the presence of greatness. He would round with only the junior most member of the team, which was thankfully me at that time, and on rounds, he would ask all about my life and interests. He learned early on of my passion for the basic sciences and that I had not completed a PhD in medical school that I had initially set out to do- a source of regret and a lost opportunity for me. He asked about my family, my hobbies, and my ambitions. In the words of Walt Whitman … he was “curious, not judgmental”. My first case with him was a cervical lymph node excision for a patient with post-transplant lymphoproliferative disorder. He donned a patriotic U.S.A. surgical cap and had me do the subcuticular stitch after the excision. Classical music was in the background and I was in rhythm … until the last stitch which I promptly and accidentally cut through the knot and the wound unraveled. I was sure this would stain my future surgical career. He on the other hand, responded with the grace that I would later realize was his signature. He comforted me and said “it is not what happens to us it is how we respond … call for a new stitch and start over”.

Over the course of my early days with Tony, he worked tirelessly to make sure I was set up for a future in the field. He reached out to Sir Peter Morris and Professor Kathryn Wood on my behalf to introduce the idea of pursuing a Doctor of Philosophy at Oxford University and sat with me for months editing, line by line, various grants so that I may secure funding for this opportunity. I would sit in his office as he would take calls from Drs. Joseph Murray and Thomas Starzl. He would finish his conversation, hang up the phone, and re-direct his undivided attention to an intern from Irmo, South Carolina interested in a career in transplantation. After 4 failed grant attempts, the 5th hit from the American Society of Transplantation and this transplant surgeon was born from the nurturing support of Dr. Anthony Monaco.


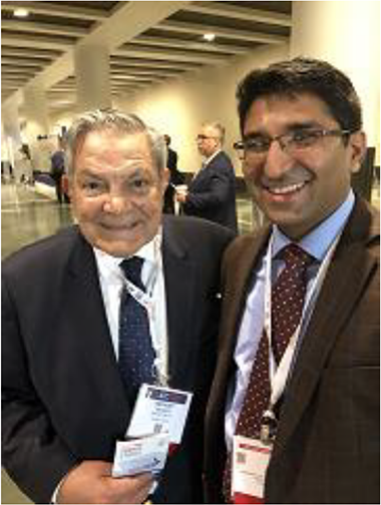
Yet, as I learned from so many since … my story was not unique. He seemed to be a mentor to so many and each received the same undivided attention. Leaders in the field including, but not limited to, Mark Hardy (his first fellow), A. Benedict Cosimi, Manikkam Suthanthiran, Martha Pavlakis, Douglas Hanto, Elizabeth Pomfret, James Pomposelli, and Xian Li were touched by Dr. Monaco. The loss of a larynx did not quiet his presence. His soft, gentle prodding and ability to make you believe you could be better than what you imagine was unparalleled. Yes, he reached the greatest heights in his career, but what's more is that he served as the platform from which a whole generation of leaders lifted off.

Sixteen years after I first met Tony, he remained present in my life as he did for the entirety of his mentee clan. Finally, it was *his* email that encouraged the leap in my own career to take on a programmatic leadership role. His salient and persuasive point hinged on the fact that it was our duty to try and reach as many people as possible for the betterment of *our* field.

Dr. Anthony Monaco will, no doubt, be remembered for his work on anti-lymphocyte globulin and the development of many transplant programs along with the litany of discoveries and breakthroughs which we enjoy as mainstream today. Yet his legacy will carry on for one reason alone … the score of people that he inspired to go on and serve as role models for future generations.

## Data Availability

The original contributions presented in the study are included in the article/Supplementary Material, further inquiries can be directed to the corresponding author.
